# Supplementary material for: Computational analysis of single-cell transcriptomics data elucidates the stabilization of Oct4 expression in the E3.25 mouse preimplantation embryo
Source: Sci Rep. 2019 Jun 20;9:8930. doi: 10.1038/s41598-019-45438-y (PMC6586892; doi:10.1038/s41598-019-45438-y)
Supplement: Supplementary file 1 — Supplementary Material [file 41598_2019_45438_MOESM1_ESM.pdf]

Supplementary Material:

Computational analysis of single-cell transcriptomics data elucidates the stabilization of Oct4 expression in the E3.25 mouse preimplantation embryo

Daniela Gerovska[1,2] and Marcos J. Araúzo-Bravo[1,2,3,4*]

[1]Computational Biology and Systems Biomedicine Group, Biodonostia Health Research Institute, Calle Doctor Beguiristain s/n, 20014 San Sebastián, Spain
[2]Computational Biomedicine Data Analysis Platform, Biodonostia Health Research Institute, Calle Doctor Beguiristain s/n, 20014 San Sebastián, Spain
[3]IKERBASQUE, Basque Foundation for Science, Calle María Díaz Harokoa 3, 48013 Bilbao, Spain
[4]CIBER of Frailty and Healthy Aging (CIBERfes), Madrid, Spain
*Corresponding author: Tel.: +34 943 00 6108, email: mararabra@yahoo.co.uk

Supplementary results

Dppa2 is highly expressed in E3.25-LNCs

The DEGs for the highly expressed transcripts in E3.25-LNC (E3.25 LNC-h-DEG) are given in **Fig. S2**. *Dppa2,* the developmental pluripotency associated 2 gene, is among the few E3.25 LNC-h-DEGs found. *Dppa2* has a DNA binding motif involved in chromatin organization, and binding sites for POU domains and Sox proteins (Aravind & Koonin, 2000)*. Dppa2* shows a noteworthy oscillatory behavior, highly expressed in all 32-34-, and 49-cell embryos, but lower expressed in 41- and 50-cell embryos. Violin plots of the transcriptomics expression from oocyte to early postimplantation stages (**Fig. S5**) show that *Dppa2* starts with a very low expression in oocytes, which grows to reach a plateau at the 8-cell stage, and (with the exception of the aforementioned 41- and 50-cell embryos) remains stable during all following preimplatation stages. *Dppa*2 is related to the regulation of histone methylation and targets Nkx2-5 and Syce1. Interestingly, the violin plots in **Fig. S5** show some correlation of the expression of *Syce1* with *Dppa2* at E3.25, where *Syce1* after an expression spike in the 34-cell embryos goes down in the 41-cell embryos, following *Dppa2*, and splits its expression into two groups of cells, one, more populated, remaining lowly expressed, and another, less populated, following the higher expression of *Dppa2*. However, in the *Nkx2-5* case there is no expression change across all the developmental stage (**Fig. S5**).

Supplementary figures

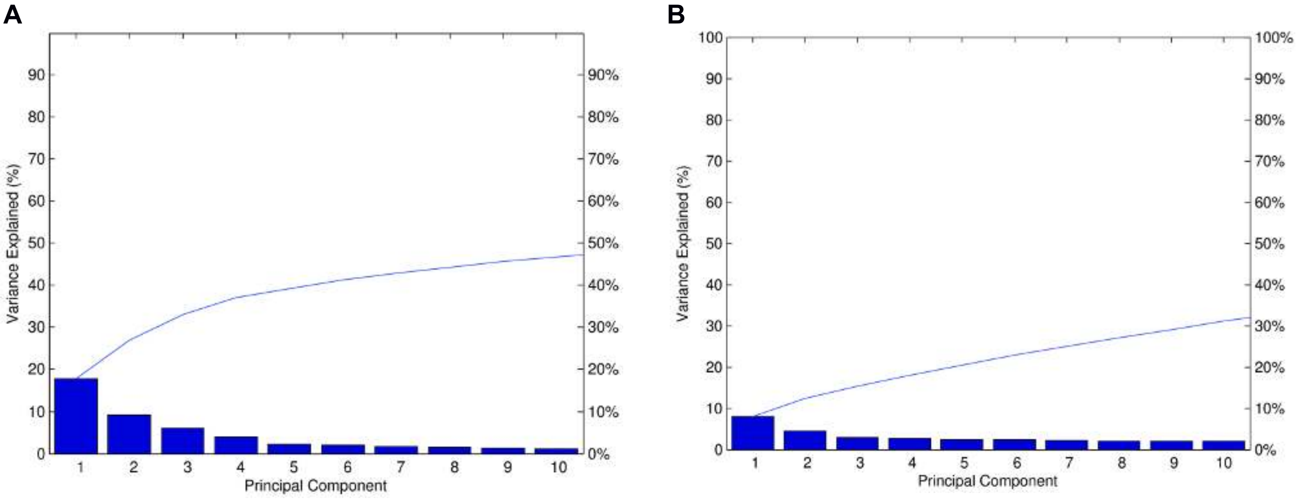

Figure S1. **Pareto charts of the PCA**. (A) All samples from **Table 1**. (B) E3.25 and E3.5 ICM single-cell data from Ohnishi *et al*. (2014). Each bar represents the percentage of variance explained by each principal component. The line above the bars shows the cumulative percentage of variance explained.

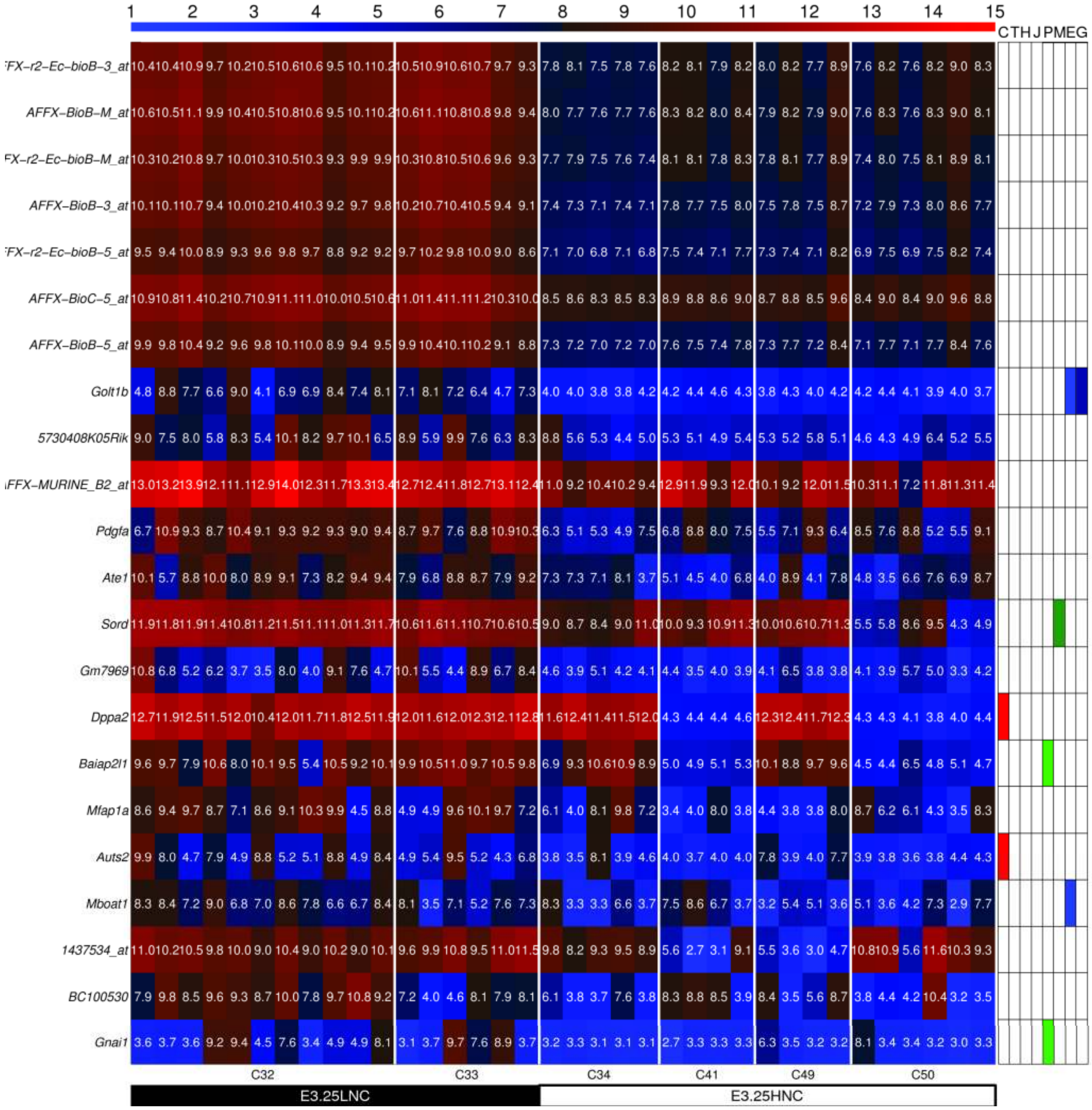

Figure S2. Highly expressed transcripts in E3.25-LNCs (E3.25 LNC-h-DEGs). Heatmap of the expression of the E3.25 LNC-h-DEGs in decreasing order of significance. The color bar codifies the gene expression in $\log_2$ scale. Higher gene expression corresponds to redder color. The table to the right annotates GO terms: C (Chromatin remodelers), T (Transcription factor activity), H (Hypoxia), J (Cell junction), P (Plasma membrane), M (Mitochondrion), E (Endoplasmatic reticulum), G (Golgi apparatus).

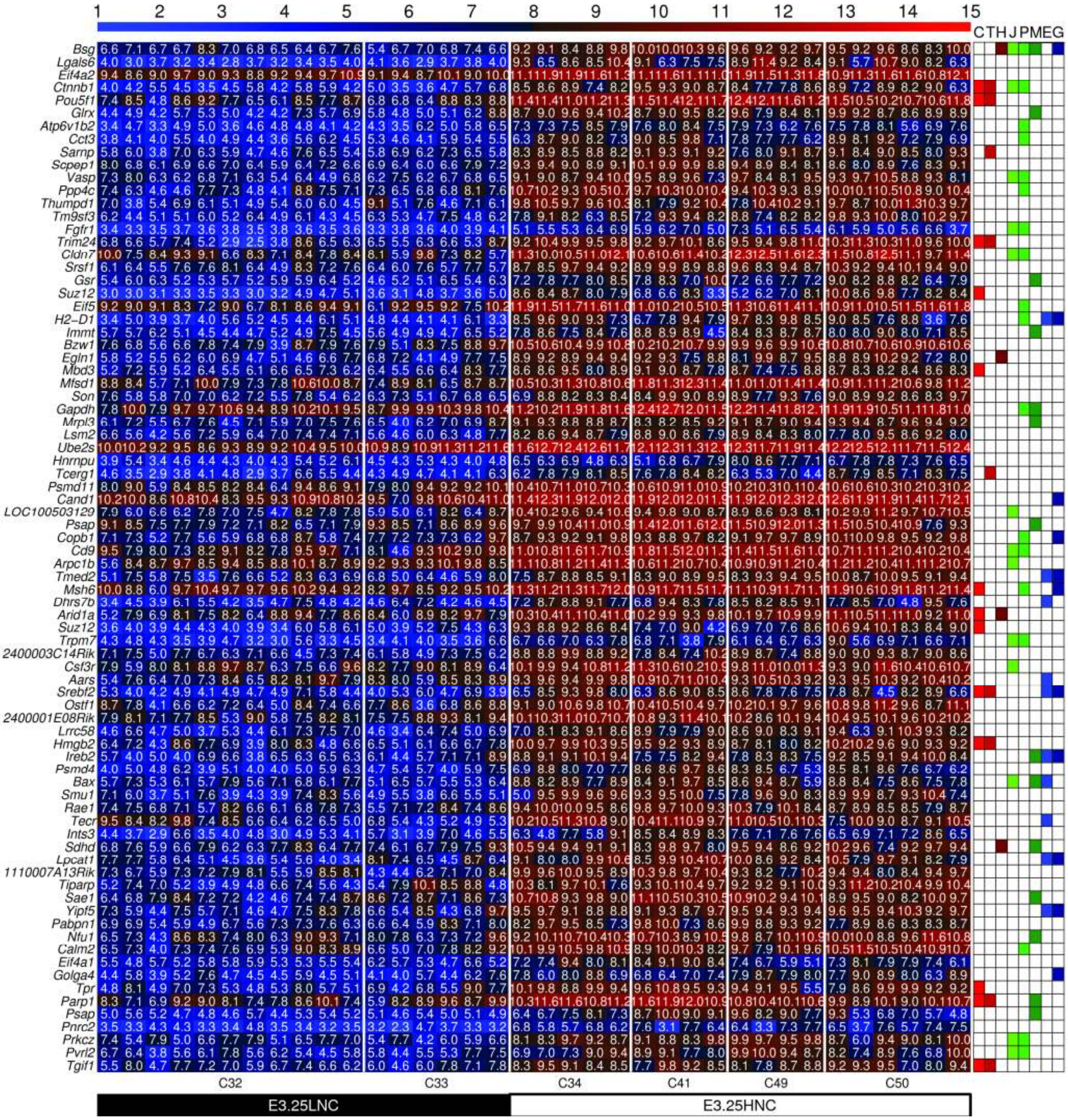

Figure S3. Highly expressed transcripts in E3.25-HNCs (E3.25 HNC-h-DEGs). Heatmaps of the expression of the 399 E3.25 HNC-h-DEGs in decreasing order of significance. The color bar codifies the gene expression in $\log_2$ scale. Higher gene expression corresponds to redder color. The table to the right annotates GO terms: C (Chromatin remodelers), T (Transcription factor activity), H (Hypoxia), J (Cell junction), P (Plasma membrane), M (Mitochondrion), E (Endoplasmatic reticulum), G (Golgi apparatus). This heatmap and the following ones represent the expression of the 399 transcripts distributed into 5 heatmaps (4 with 80 probes and the last one with 79 transcripts).

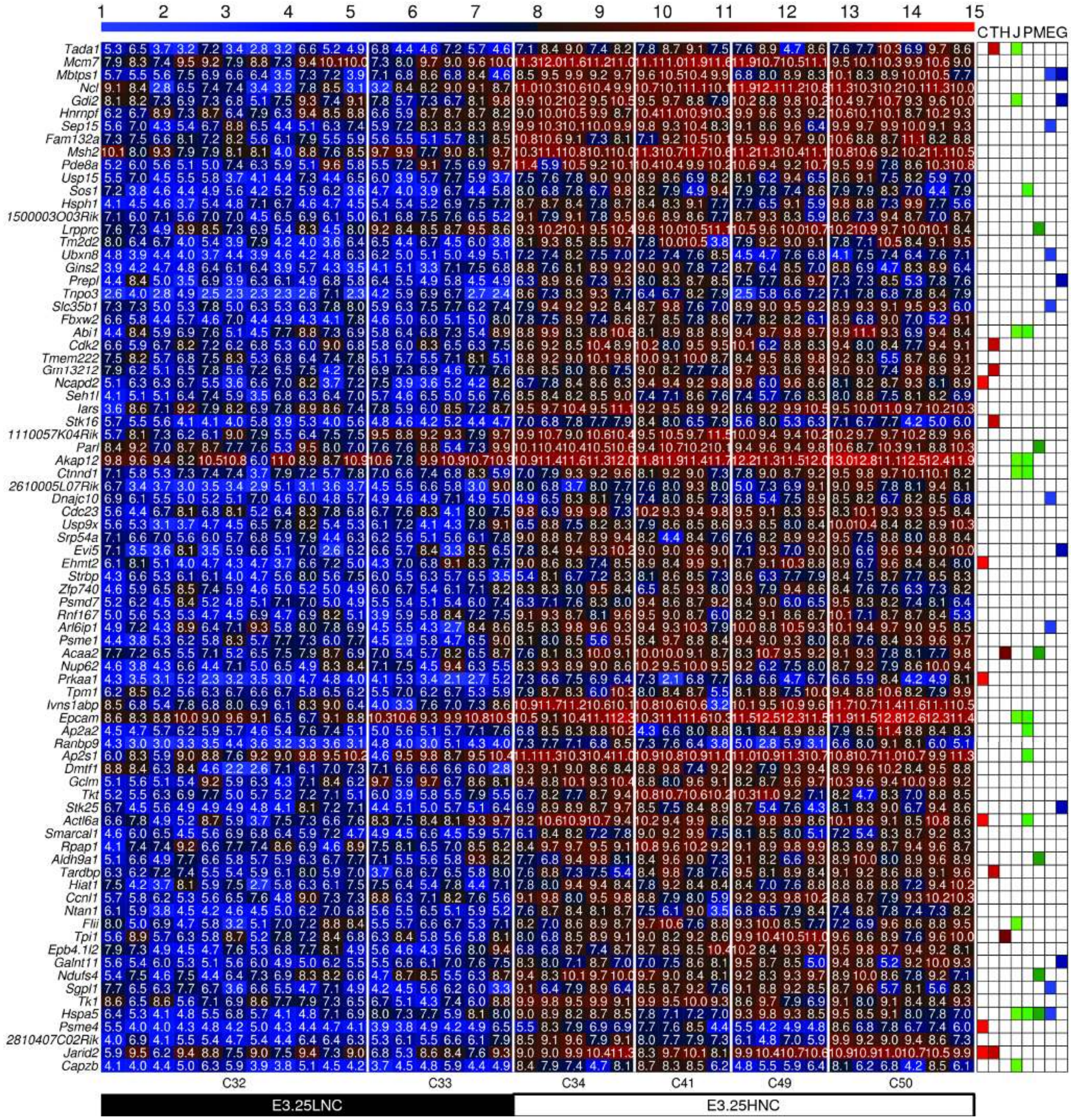

Figure S3 continuation (2/5)

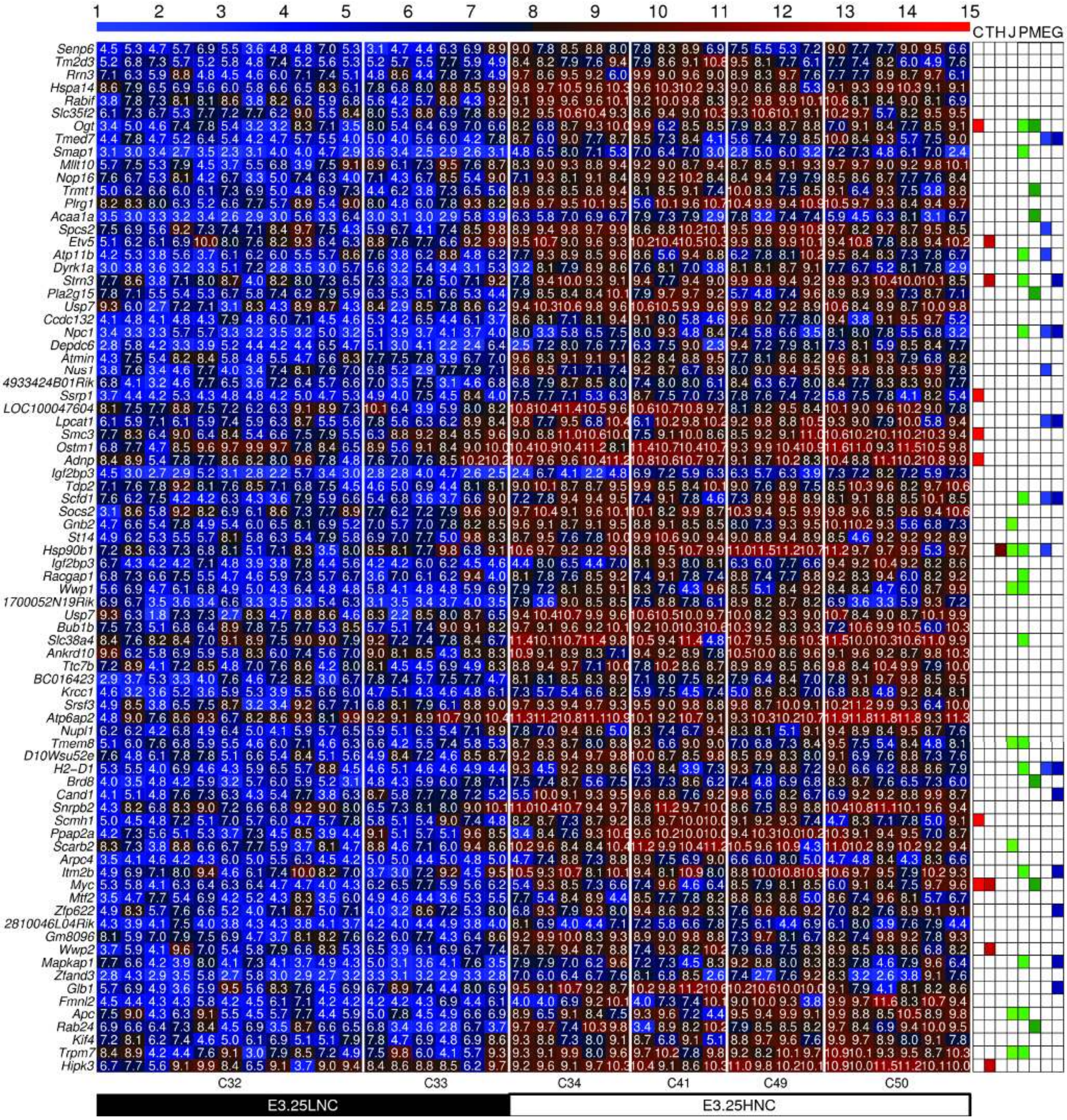

Figure S3 continuation (3/5)

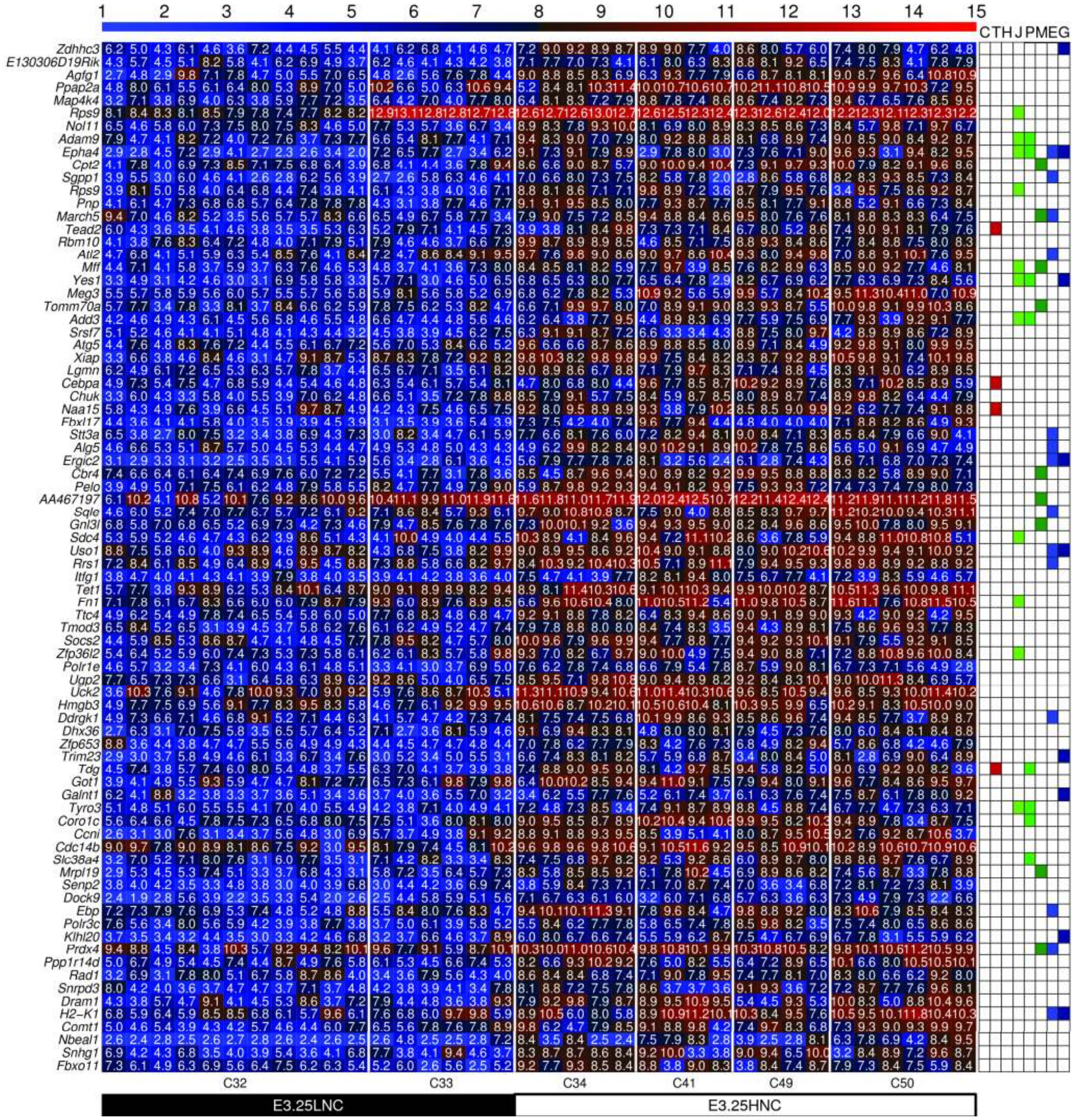

Figure S3 continuation (4/5)

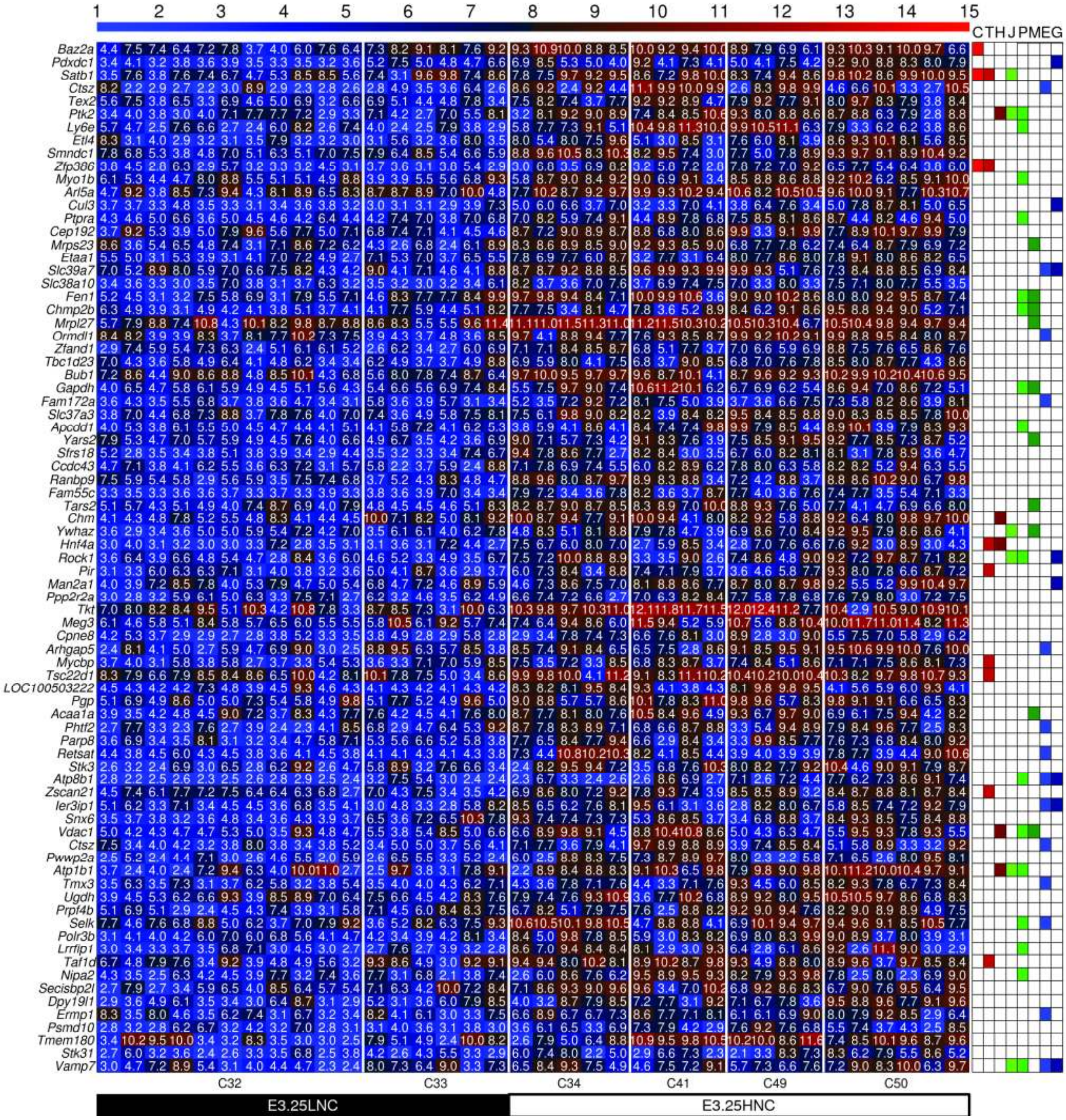

Figure S3 continuation (5/5)

LOC100503129. ***Ywhaq***, tyrosine 3-monooxygenase/tryptophan 5-monooxygenase activation protein, theta polypeptide
LOC10004706. proteasome assembly chaperone 2-like
1110007A13Rik: ***Mcmbp***, MCM (minichromosome maintenance deficient) binding protein
1110057K04Rik: ***Ldah***, lipid droplet associated hydrolase
1500003O03Rik: ***Chp1***, calcineurin-like EF hand protein 1
1700052N19Rik: ***Armt1***, acidic residue methyltransferase 1
2400001E08Rik: ***Lamtor1***, late endosomal/lysosomal adaptor, MAPK and MTOR activator 1
2400003C14Rik: ***Ist1***, increased sodium tolerance 1 homolog (yeast)
2610005L07Rik: Also known as *2810038F24Rik* and *6720476A01Rik*
2810046L04Rik: ***Proser1***, proline and serine rich 1
2810407C02Rik: ***Selt***, selenoprotein T
4933424B01Rik: ***Asun***, asunder, spermatogenesis regulator

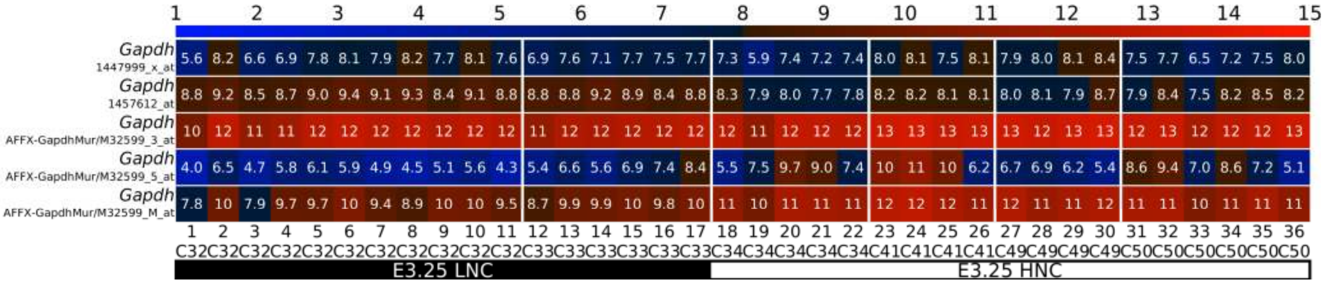

Figure S4. Heatmap of the expression of the five probes targeting *Gapdh* in the single cells from E3.25 of Ohnishi *et al.* (2014). The color bar codifies the gene expression in log_2 scale. Higher gene expression corresponds to redder color.

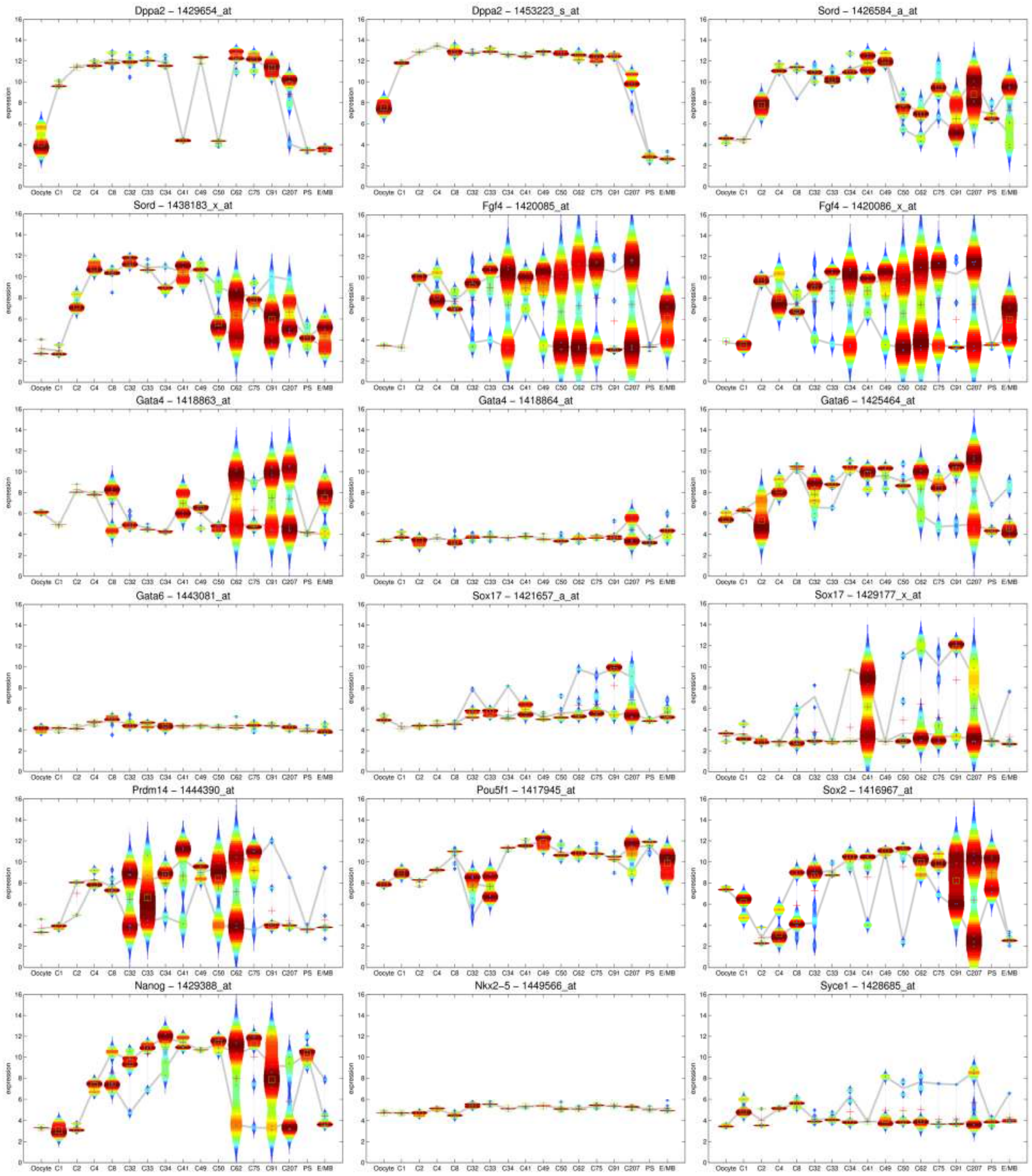

Figure S5. Violin plots of the distribution of the expression of different characteristic genes across the developmental stages. The mean and median are shown as red crosses and green squares, respectively. The data points are over-imposed in light blue. The official gene name and the Affymetrix identifier are on the top of each plot.

Molecular-function

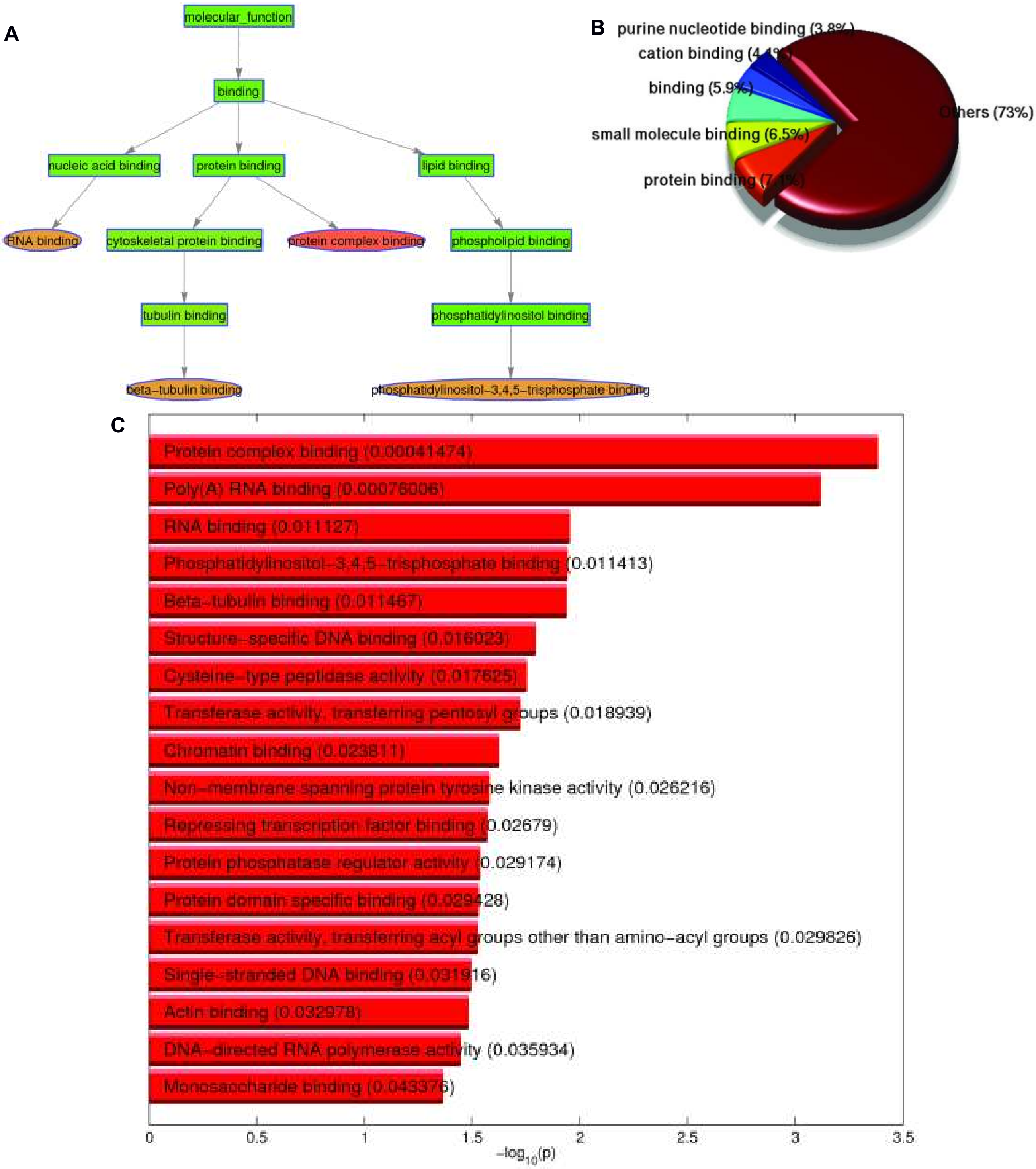

Figure S6. GO enrichment analysis of E3.25 HNC-h-DEGs. (A) Direct acyclic graph (DAG) associated to the top five significantly enriched gene ontology terms. Higher term significance is marked by redder box framing the term. The significant enriched terms are encircled by ellipses. (B) Pie graph. (C) Bar plot of the $-\log_{10}(p)$ of the significantly enriched terms. Longer bars denote higher statistical significance of the enrichment (the p-values are in parentheses).

Biological-processes

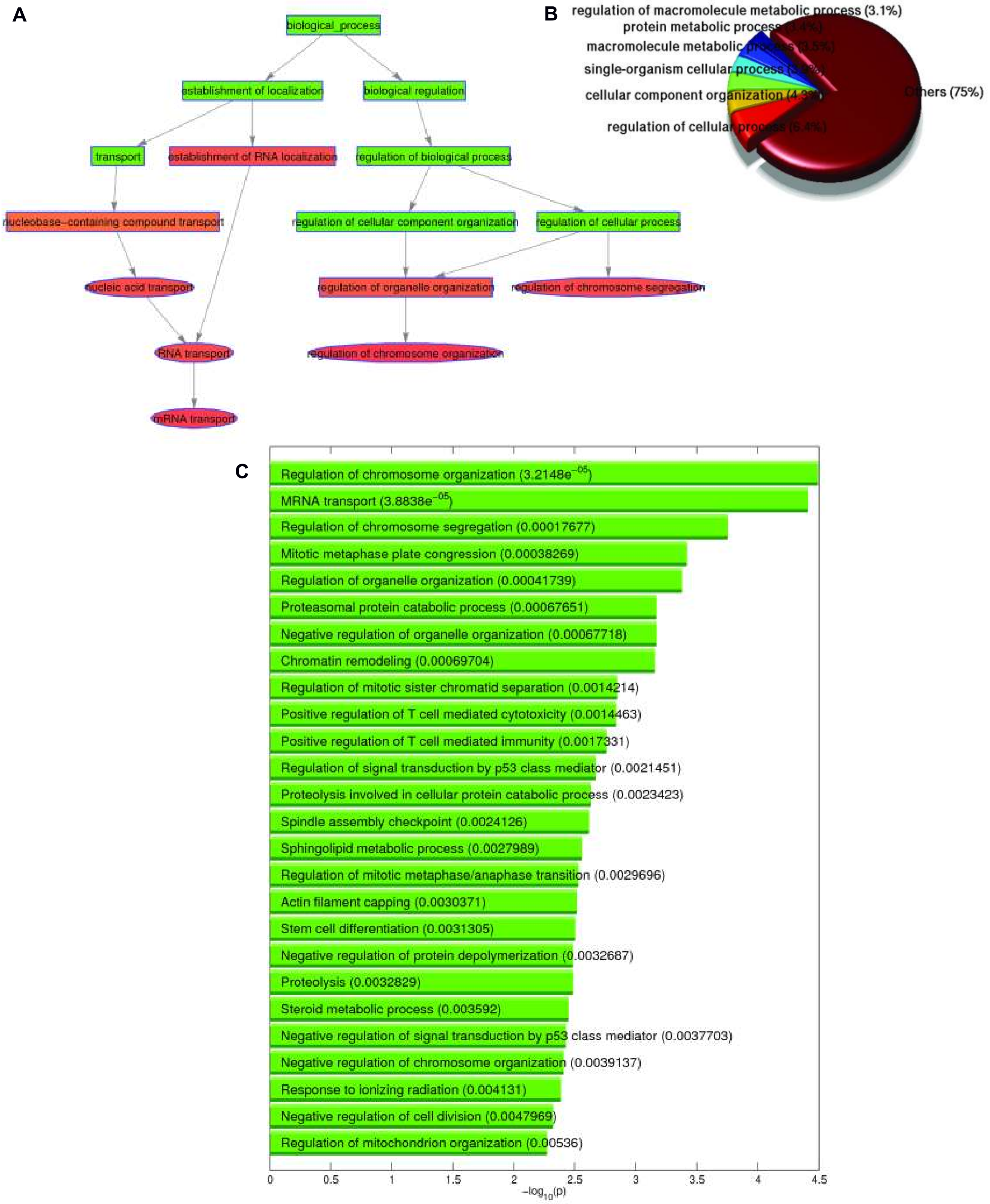

Figure S6 continuation (2/3)

Cellular-components

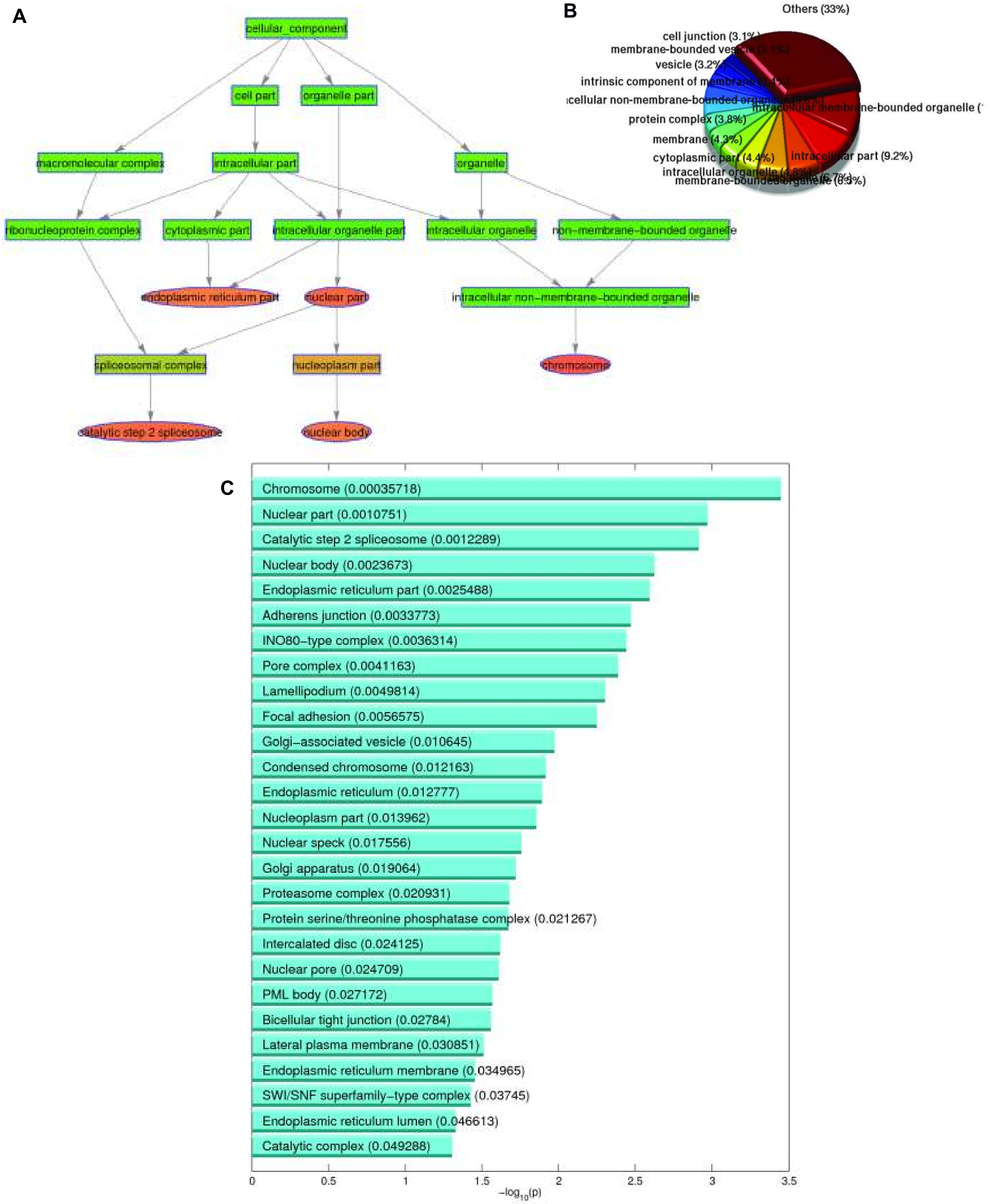

Figure S6 continuation (3/3)

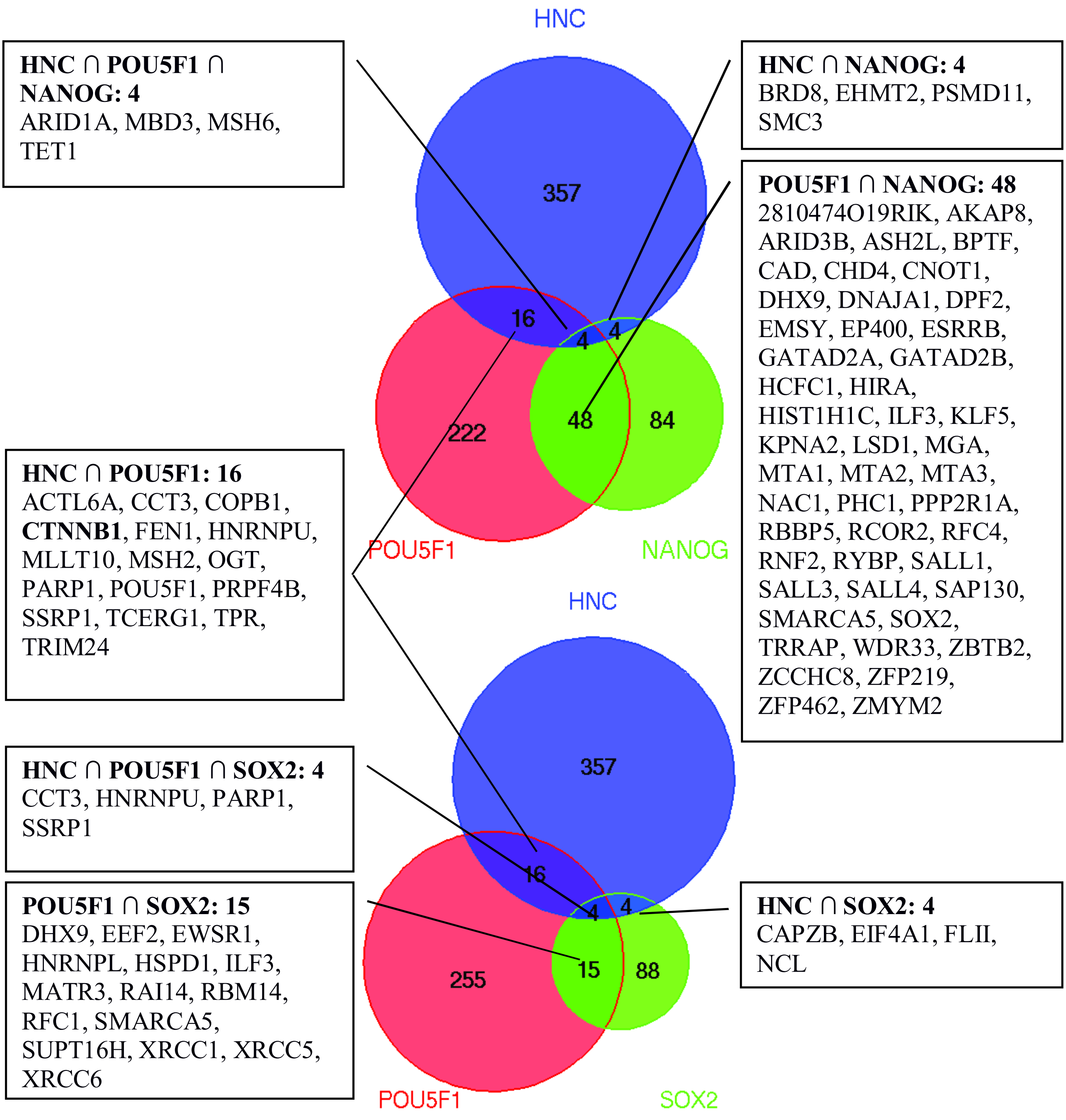

Figure S7. **Euler-Venn diagram of the intersections of the E3.25 HNC-h-DEGs with the POU5F1, NANOG and SOX2 interactomes.**

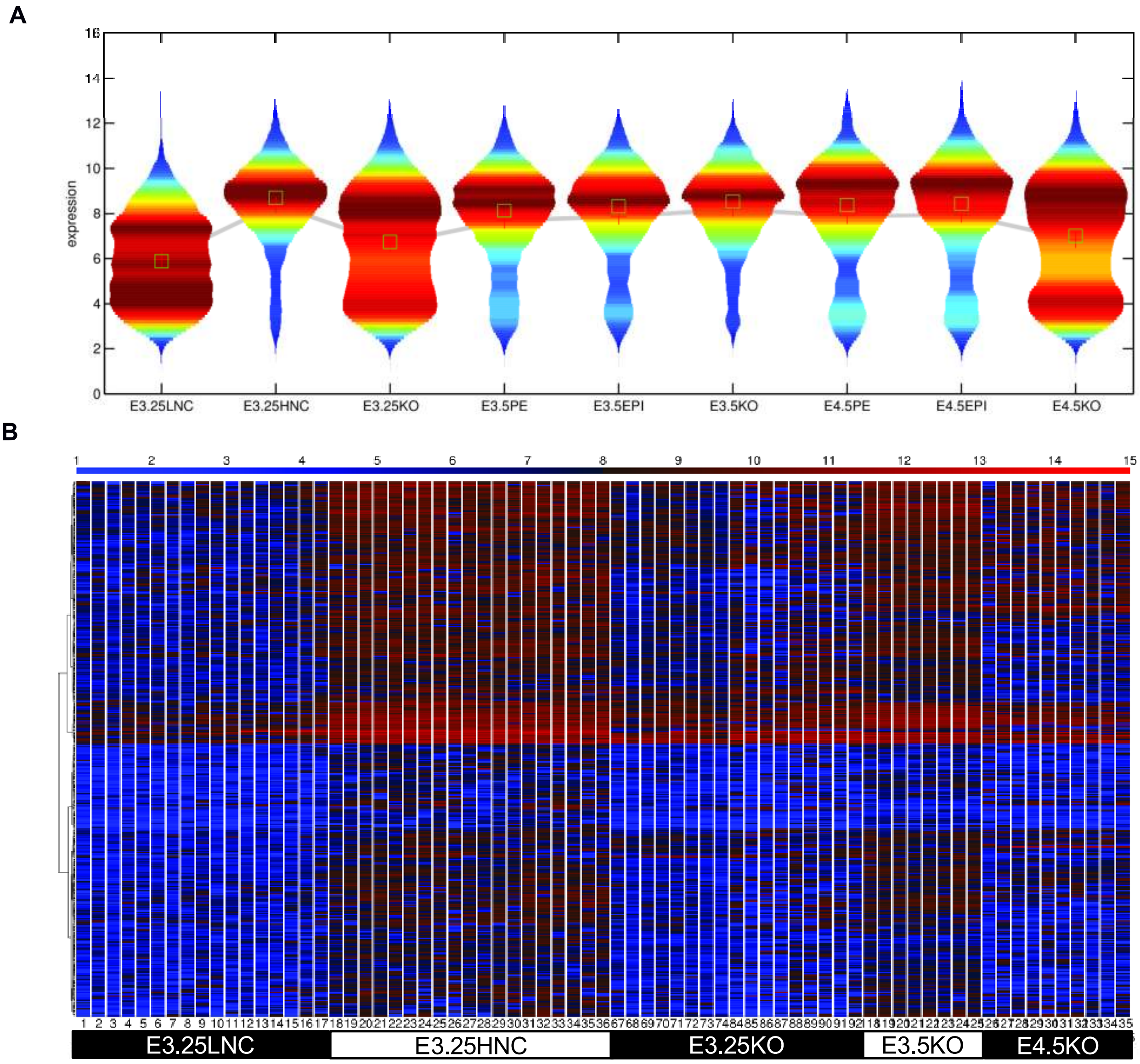

Figure S8. E3.25 HNC-h-DEGs across wild type and *Fgf4*-KO samples. (A) Violin plots. (B) Heatmap.

L32ICM ∩ POU5F1 ∩ NANOG: 5
CNOT1, ESRRB, MBD3, SALL4, SOX2

L32ICM ∩ NANOG: 11
CNOT1, ESRRB, EZH2, MBD3, MLL3, PUM1, SALL4, SGOL2, SOX2, UBTF, UCHL5

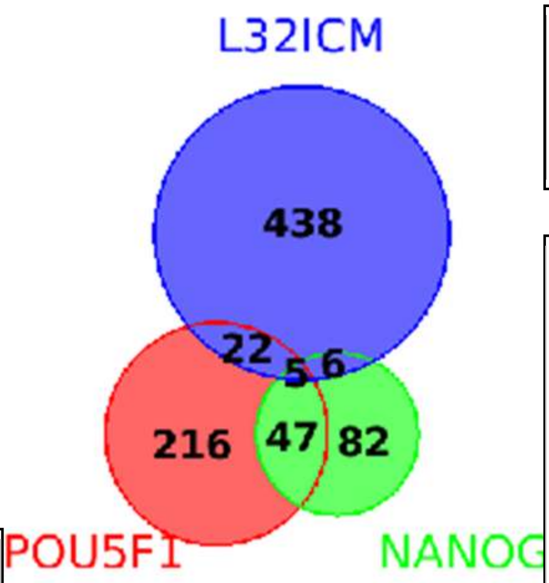

POU5F1 ∩ NANOG: 52
2810474O19RIK, AKAP8, ARID1A, ARID3B, ASH2L, BPTF, CAD, CHD4, CNOT1, DHX9, DNAJA1, DPF2, EMSY, EP400, ESRRB, GATAD2A, GATAD2B, HCFC1, HIRA, HIST1H1C, ILF3, KLF5, KPNA2, LSD1, MBD3, MGA, MSH6, MTA1, MTA2, MTA3, NAC1, PHC1, PPP2R1A, RBBP5, RCOR2, RFC4, RNF2, RYBP, SALL1, SALL3, SALL4, SAP130, SMARCA5, SOX2, TET1, TRRAP, WDR33, ZBTB2, ZCCHC8, ZFP219, ZFP462, ZMYM2

L32ICM ∩ POU5F1: 27
0610010K14RIK, ACIN1, BRD4, CDC73, CMAS, CNOT1, CTBP2, ESRRB, GNB1, HDAC2, HNRNPAB, HNRNPU, KLF4, L1TD1, MBD3, PCBP2, POU5F1, PPP1CC, RAD21, RTN4, SALL4, SOX2, SSRP1, SUDS3, TRIM24, WAPAL, ZBTB10

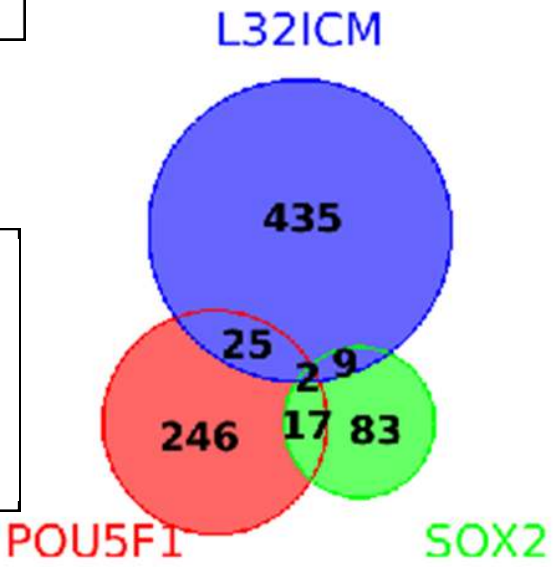

L32ICM ∩ SOX2: 11
DDX17, H2AFY, HNRNPA3, HNRNPC, HNRNPU, KHDRBS1, LACTB, PABPC4, RPL35A, SSBP1, SSRP1

POU5F1 ∩ SOX2: 19
CCT3, DHX9, EEF2, EWSR1, HNRNPL, HNRNPU, HSPD1, ILF3, MATR3, PARP1, RAI14, RBM14, RFC1, SMARCA5, SSRP1, SUPT16H, XRCC1, XRCC5, XRCC6

L32ICM ∩ POU5F1 ∩ SOX2: 2
HNRNPU, SSRP1

Figure S9. Euler-Venn diagram of the intersections of the L32ICM-h-DEGs with the POU5F1, NANOG and SOX2 interactomes. The common genes between the same type of intersections as in the E3-25-HNC case in **Fig. S7**, are highlighted in red.

Supplementary tables

Table S1. Significant GO enriched molecular functions of the E3.25 HNC-h-DEGs.

Count Ratio	*p*-val	GO Id	GO Term	Genes
44/670	0.00041474	GO:0032403	protein complex binding	*Abi1 Actl6a Adam9 Ap1ar Ap2a2 Apc Arid1a Arpc1b Arpc4 Atp1b1 Bax Capzb Cd9 Cdk2 Cebpa Clasp1 Coro1c Epcam Fn1 Gapdh Gnb2 H2-D1 H2-K1 Kat5 Kif4 Mbd3 Msh2 Msh6 Myc Myo1b Ppp2r2a Ppp4c Ptk2 Ptpra Racgap1 Rbm10 Smc3 Strn3 Tmod3 Tpm1 Tpr Tsn Vdac1 Ywhaz*
58/975	0.00076006	GO:0044822	poly(A) RNA binding	*Acaa2 Bzw1 Cct3 Csde1 Dhx36 Eif4a1 Eif4a2 Eif5 Gdi2 Gnl3l H2-D1 H2-K1 Hmgb2 Hnrnpf Hnrnpu Igf2bp3 Immt Ireb2 Lrpprc Lsm2 Mrpl27 Mrpl3 Mrps23 Naa15 Ncl Nol11 Nop16 Pabpn1 Parp1 Pou5f1 Prpf4b Psmd4 Rbm10 Rbm12 Rps9 Rrs1 Sarnp Secisbp2l Smndc1 Snrpd3 Son Srp54a Srsf1 Srsf3 Srsf7 Ssrp1 Strbp Tardbp Tcerg1 Thumpd1 Tpr Trmt1 Tsn Uso1 Yars2 Ywhaz Zfp36l2 Zfp622*
65/1263	0.011127	GO:0003723	RNA binding	*Aars Acaa2 Baz2a Bzw1 Cct3 Csde1 Dhx36 Eif4a1 Eif4a2 Eif5 Gdi2 Gnl3l H2-D1 H2-K1 Hmgb2 Hnrnpf Hnrnpu Hsp90b1 Igf2bp3 Immt Ireb2 Lrpprc Lsm2 Mrpl27 Mrpl3 Mrps23 Naa15 Ncl Nol11 Nop16 Pabpn1 Parp1 Pou5f1 Prpf4b Psmd4 Rbm10 Rbm12 Rbm25 Rps9 Rrs1 Sarnp Secisbp2l Slu7 Smndc1 Snrpb2 Snrpd3 Son Srp54a Srsf1 Srsf3 Srsf7 Ssrp1 Strbp Suz12 Tardbp Tcerg1 Thumpd1 Tpr Trmt1 Tsn Uso1 Yars2 Ywhaz Zfp36l2 Zfp622*
4/27	0.011413	GO:0005547	phosphatidylinositol-3,4,5-trisphosphate binding	*Mapkap1 Myo1b Ogt Racgap1*
4/27	0.011467	GO:0048487	beta-tubulin binding	*Adnp Capzb Lrpprc Racgap1*
14/197	0.016023	GO:0043566	structure-specific DNA binding	*Actl6a Ctnnb1 Dhx36 Lrpprc Mbd3 Mcm7 Msh2 Msh6 Myc Tardbp Tdg Tdp2 Tet1 Tsn*
10/126	0.017625	GO:0008234	cysteine-type peptidase activity	*Capn7 Ctsz Ermp1 Lgmn Senp2 Senp6 Spcs2 Usp15 Usp7 Usp9x*
4/31	0.018939	GO:0016763	transferase activity, transferring pentosyl groups	*Parp1 Parp8 Pnp Tiparp*
22/367	0.023811	GO:0003682	chromatin binding	*Actl6a Adnp Arid1a Ctnnb1 Dmtf1 Hmgb2 Hmgb3 Jarid2 Kat5 Mbd3 Msh6 Pou5f1 Prkaa1 Satb1 Smc3 Srebf2 Ssrp1 Suz12 Tgif1 Tpr Trim24 Zfp386*
4/34	0.026216	GO:0004715	non-membrane spanning protein tyrosine kinase activity	*Dyrk1a Ptk2 Stk16 Yes1*
5/49	0.02679	GO:0070491	repressing transcription factor binding	*Ctnnb1 Hnf4a Kat5 Myc Tcerg1*
4/35	0.029174	GO:0019888	protein phosphatase regulator activity	*Ppp1r14d Ppp2r2a Ppp2r5c Ubxn2a*
28/501	0.029428	GO:0019904	protein domain specific binding	*Adam9 Bax Calm2 Capn7 Cebpa Chmp2b Ctnnd1 Cul3 Ehmt2 Epb4.1l2 Epha4 Hmgb2 Hspa5 Nup62 Ogt Ostf1 Pou5f1 Prkcz Ptk2 Sarnp Senp2 Son Sos1 Srsf1 Strn3 Tdg Vasp Ywhaz*
11/155	0.029826	GO:0016747	transferase activity, transferring acyl groups other than amino-acyl groups	*Acaa1a Acaa2 Cpt2 Crot Kat5 Lpcat1 Naa15 Ogt Pla2g15 Tada1 Zdhhc3*
5/51	0.031916	GO:0003697	single-stranded DNA binding	*Lrpprc Mcm7 Msh2 Tdp2 Tsn*
16/255	0.032978	GO:0003779	actin binding	*Add3 Arpc1b Arpc4 Capzb Coro1c Epb4.1l2 Flii Fmnl2 Gsn Klhl20 Myo1b Ptk2 Tmod3 Tpm1 Trpm7 Vasp*
4/37	0.035934	GO:0003899	DNA-directed RNA polymerase activity	*Polr1e Polr3b Polr3c Rpap1*
5/55	0.043376	GO:0048029	monosaccharide binding	*Bsg Egln1 Ogt Tkt Ugp2*

Table S2. Significant GO enriched biological-processes of the E3.25 HNC-h-DEGs.

Count Ratio	*p*-val	GO id	GO term	Genes
17/138	3.21e-05	GO:0033044	regulation of chromosome organization	*Apc Atg5 Bub1 Bub1b Cep192 Ctnnb1 Cul3 Dhx36 Ehmt2 Jarid2 Mtf2 Myc Ogt Pou5f1 Senp6 Tet1 Tpr*
12/77	3.88e-05	GO:0051028	mRNA transport	*Igf2bp3 Lrpprc Nup62 Nupl1 Pabpn1 Samp Seh1l Senp2 Srsf1 Srsf3 Srsf7 Tpr*
8/43	0.000177	GO:0051983	regulation of chromosome segregation	*Apc Bub1 Bub1b Cep192 Ctnnb1 Cul3 Racgap1 Tpr*
4/11	0.000382	GO:0007080	mitotic metaphase plate congression	*Cdc23 Cul3 Rrs1 Seh1l*
39/543	0.000417	GO:0033043	regulation of organelle organization	*Acaa2 Ap1ar Apc Arpc1b Arpc4 Atg5 Bax Bub1 Bub1b Capzb Cdc23 Cep192 Clasp1 Ctnnb1 Cul3 Dhx36 Ehmt2 Gsn Hsph1 Jarid2 Lrpprc March5 Msh2 Mtf2 Myc Ogt Parl Pmaip1 Pou5f1 Psmd10 Racgap1 Rad1 Sdc4 Senp6 Tet1 Tmod3 Tpm1 Tpr Vasp*
17/177	0.000677	GO:0010498	proteasomal protein catabolic process	*Apc Bub1b Cul3 Dnajc10 Hsp90b1 Kat5 Klhl20 Ppp2r5c Psme4 Ube2s Ubxn2a Ubxn8 Usp15 Usp7 Usp9x Wwp1 Wwp2*
17/177	0.000677	GO:0010639	negative regulation of organelle organization	*Acaa2 Apc Atg5 Capzb Cep192 Clasp1 Gsn Jarid2 Msh2 Mtf2 Parl Psmd10 Rad1 Tet1 Tmod3 Tpm1 Tpr*
11/90	0.000697	GO:0006338	chromatin remodeling	*Actl6a Arid1a Baz2a Hmgb2 Hmgb3 Mbd3 Myc Psme4 Satb1 Scmh1 Ssrp1*
5/24	0.00142	GO:0010965	regulation of mitotic sister chromatid separation	*Apc Bub1b Cep192 Cul3 Tpr*
4/15	0.00145	GO:0001916	positive regulation of T cell mediated cytotoxicity	*H2-D1 H2-K1 Pnp Pvrl2*
5/25	0.00173	GO:0002711	positive regulation of T cell mediated immunity	*H2-D1 H2-K1 Pnp Prkcz Pvrl2*
6/37	0.00215	GO:1901796	regulation of signal transduction by p53 class mediator	*Dyrk1a Pmaip1 Psmd10 Rrn3 Senp2 Trim24*
22/283	0.00234	GO:0051603	proteolysis involved in cellular protein catabolic process	*Apc Bub1b Cul3 Dnajc10 Hsp90b1 Hspa5 Kat5 Klhl20 Lgmn Ppp2r5c Psmd11 Psmd4 Psme4 Sae1 Ube2s Ubxn2a Ubxn8 Usp15 Usp7 Usp9x Wwp1 Wwp2*
4/17	0.00241	GO:0071173	spindle assembly checkpoint	*Apc Bub1 Cep192 Tpr*
8/64	0.00280	GO:0006665	sphingolipid metabolic process	*B4galt6 Bax Ormdl1 Pla2g15 Ppap2a Psap Sgpl1 Sgpp1*
5/28	0.00297	GO:0030071	regulation of mitotic metaphase/anaphase transition	*Apc Cdc23 Cep192 Cul3 Tpr*
4/18	0.00304	GO:0051693	actin filament capping	*Capzb Gsn Tmod3 Tpm1*
7/52	0.00313	GO:0048863	stem cell differentiation	*Epcam Fgfr1 Jarid2 Mtf2 Pou5f1 Psmd11 Zfp281*
6/40	0.00326	GO:1901880	negative regulation of protein depolymerization	*Apc Capzb Clasp1 Gsn Tmod3 Tpm1*
36/550	0.00328	GO:0006508	proteolysis	*Adam9 Apc Atp6ap2 Bub1b Capn7 Ctsz Cul3 Dnajc10 Enpep Ermp1 Hsp90b1 Hspa5 Kat5 Klhl20 Lgmn Mbtps1 Parl Ppp2r5c Prepl Psmd11 Psmd4 Psme4 Sae1 Scpep1 Senp2 Senp6 Spcs2 St14 Ube2s Ubxn2a Ubxn8 Usp15 Usp7 Usp9x Wwp1 Wwp2*
12/125	0.00359	GO:0008202	steroid metabolic process	*Acaa1a Atp8b1 Cebpa Ebp Mbtps1 Npc1 Prkaa1 Sgpl1 Sqle Srebf2 Tecr Tiparp*
4/19	0.00377	GO:1901797	negative regulation of signal transduction by p53 class mediator	*Dyrk1a Psmd10 Rrn3 Senp2*
7/54	0.00391	GO:2001251	negative regulation of chromosome organization	*Apc Atg5 Cep192 Jarid2 Mtf2 Tet1 Tpr*
9/82	0.00413	GO:0010212	response to ionizing radiation	*Bax Ints3 Kat5 Msh2 Myc Pmaip1 Pnp Prkaa1 Rad1*
6/43	0.00480	GO:0051782	negative regulation of cell division	*Apc Cep192 Msh2 Myc Rad1 Tpr*
7/57	0.00536	GO:0010821	regulation of mitochondrion organization	*Acaa2 Bax Lrpprc March5 Parl Pmaip1 Psmd10*

Table S3. Significant GO enriched cellular-components of the E3.25 HNC-h-DEGs.

Count Ratio	*p*-val	GO id	GO term	Genes
25/298	0.000357	GO:0005694	chromosome	*Add3 Bub1 Bub1b Cdk2 Clasp1 Dhx36 Ehmt2 Fbxo11 Hmgb2 Hmgb3 Kif4 Lrpprc Mbd3 Msh2 Msh6 Ncapd2 Ppp2r5c Prpf4b Rad1 Rrs1 Seh1l Smc3 Ssrp1 Suz12 Tpr*
91/1628	0.00108	GO:0044428	nuclear part	*Actl6a Add3 Ap1ar Apc Arid1a Baz2a Brd8 Bub1 Bub1b Ccnl1 Cdc14b Cdc23 Cdk2 Cebpa Ctnnb1 Dyrk1a Epb4.1l2 Ergic2 Fbxo11 Fen1 Gnl3l Hipk3 Hnrnpf Hnrnpu Hspa5 Ino80e Ints3 Jarid2 Kat5 Klhl20 Lrpprc Lsm2 Mbd3 Med14 Mrpl19 Mrps23 Msh2 Msh6 Mtf2 Myc Ncapd2 Ncl Nol11 Nop16 Npc1 Nup62 Nupl1 Ogt Parp1 Plrg1 Polr1e Polr3b Polr3c Pou5f1 Prkcz Prpf4b Ptk2 Rad1 Rae1 Retsat Rps9 Rrn3 Rrs1 Sae1 Samp Satb1 Seh1l Senp2 Slu7 Smarcal1 Smc3 Smndc1 Snrpb2 Snrpd3 Son Srebf2 Srsf1 Srsf3 Strn3 Suz12 Tada1 Taf1d Tc...*
9/70	0.00123	GO:0071013	catalytic step 2 spliceosome	*Hnrnpf Hnrnpu Lsm2 Plrg1 Prpf4b Slu7 Snrpb2 Snrpd3 Srsf1*
16/184	0.00237	GO:0016604	nuclear body	*Ccnl1 Cdk2 Dyrk1a Hipk3 Klhl20 Myc Ptk2 Samp Satb1 Senp2 Slu7 Son Srsf1 Srsf3 Tdg Tdp2*
23/306	0.00255	GO:0044432	endoplasmic reticulum part	*Arl6ip1 Atg5 Bax Dnajc10 Ebp H2-D1 H2-K1 Hsp90b1 Hspa5 March5 Nus1 Ormdl1 Retsat Selk Sep15 Spcs2 Srebf2 Tecr Tkt Tyro3 Ubxn8 Vamp7 Yipf5*
24/331	0.00338	GO:0005912	adherens junction	*Adam9 Akap12 Apc Arpc1b Bsg Cd9 Coro1c Ctnnb1 Ctnnd1 Epb4.1l2 Gdi2 Gnb2 Gsn Hsp90b1 Hspa5 Ptk2 Pvrl2 Rps9 Scarb2 Sdc4 Tada1 Vasp Yes1 Ywhaz*
4/19	0.00363	GO:0097346	INO80-type complex	*Actl6a Brd8 Ino80e Kat5*
7/55	0.00412	GO:0046930	pore complex	*Bax Nup62 Nupl1 Seh1l Senp2 Tpr Vdac1*
10/100	0.00498	GO:0030027	lamellipodium	*Abi1 Apc Capzb Ctnnb1 Ctnnd1 Gsn Ptk2 Rock1 Tmod3 Vasp*
21/289	0.00566	GO:0005925	focal adhesion	*Adam9 Akap12 Arpc1b Bsg Cd9 Coro1c Epb4.1l2 Gdi2 Gnb2 Gsn Hsp90b1 Hspa5 Ptk2 Pvrl2 Rps9 Scarb2 Sdc4 Tada1 Vasp Yes1 Ywhaz*
4/25	0.0106	GO:0005798	Golgi-associated vesicle	*Copb1 Scfd1 Stk16 Tmed2*
6/52	0.0122	GO:0000793	condensed chromosome	*Add3 Cdk2 Hmgb2 Lrpprc Ncapd2 Rrs1*
50/896	0.0128	GO:0005783	endoplasmic reticulum	*Alg5 Atl2 Atp11b Atp8b1 Bax Ctsz Ddrgk1 Dhrs7b Dnajc10 Ebp Epha4 Ergic2 Ermp1 Fam172a H2-D1 H2-K1 Hsp90b1 Hspa5 Ier3ip1 Ireb2 Lpcat1 March5 Mbtps1 Npc1 Nus1 Ormdl1 Phtf2 Prdx4 Retsat Rrs1 Scfd1 Selk Sep15 Sgpl1 Sgpp1 Slc35b1 Slc39a7 Spcs2 Sqle Srebf2 Stt3a Tecr Tmed2 Tmed7 Tmx3 Ubxn2a Ubxn8 Uso1 Vamp7 Yipf5*
29/469	0.0140	GO:0044451	nucleoplasm part	*Actl6a Brd8 Ccnl1 Cdk2 Dyrk1a Hipk3 Ints3 Jarid2 Kat5 Klhl20 Mbd3 Med14 Mtf2 Myc Ogt Polr3b Polr3c Ptk2 Samp Satb1 Senp2 Slu7 Son Srsf1 Srsf3 Suz12 Tada1 Tdg Tdp2*
7/71	0.0176	GO:0016607	nuclear speck	*Ccnl1 Dyrk1a Samp Slu7 Son Srsf1 Srsf3*
46/832	0.0191	GO:0005794	Golgi apparatus	*Ap1ar Atp8b1 B4galt6 Clasp1 Copb1 Csde1 Cul3 Epha4 Ergic2 Evi5 Galnt1 Galnt11 Gdi2 Glb1 Gnptab Golga4 H2-D1 H2-K1 Ier3ip1 Ireb2 Itm2b Klhl20 Lpcat1 Man2a1 Mapkap1 Mbtps1 Msh6 Npc1 Pdxdc1 Prepl Rock1 Scfd1 Slc38a10 Slc39a7 Srebf2 Stk25 Strn3 Tmed2 Tmed7 Trim23 Uso1 Vamp7 Yes1 Yipf5 Zdhhc3 Zfp622*
6/58	0.0209	GO:0000502	proteasome complex	*Psmd10 Psmd11 Psmd4 Psmd7 Psme1 Psme4*
4/30	0.0213	GO:0008287	protein serine/threonine phosphatase complex	*Ppp2r2a Ppp2r5c Ppp4c Strn3*
4/31	0.0241	GO:0014704	intercalated disc	*Atp1b1 Capzb Ctnnb1 Ptk2*
5/45	0.0247	GO:0005643	nuclear pore	*Nup62 Nupl1 Seh1l Senp2 Tpr*
6/61	0.0272	GO:0016605	PML body	*Hipk3 Klhl20 Satb1 Senp2 Tdg Tdp2*
7/77	0.0278	GO:0005923	bicellular tight junction	*Apc Cldn7 Ctnnb1 Ctnnd1 Epcam Prkcz Vasp*
4/33	0.0309	GO:0016328	lateral plasma membrane	*Apc Cldn7 Ctnnb1 Epcam*
12/168	0.0350	GO:0005789	endoplasmic reticulum membrane	*Bax Ebp Hsp90b1 Hspa5 March5 Nus1 Ormdl1 Retsat Selk Tkt Tyro3 Vamp7*
6/65	0.0375	GO:0070603	SWI/SNF superfamily-type complex	*Actl6a Arid1a Brd8 Ino80e Kat5 Mbd3*
4/37	0.0466	GO:0005788	endoplasmic reticulum lumen	*Dnajc10 Hsp90b1 Hspa5 Sep15*
36/666	0.0493	GO:1902494	catalytic complex	*Actl6a Atp1b1 Brd8 Cand1 Cdc23 Cdk2 Chm Chuk Cul3 Gclm Gnb2 Ino80e Jarid2 Kat5 Klhl20 Mbd3 Mtf2 Naa15 Ndufs4 Ogt Polr1e Polr3b Polr3c Ppp2r2a Ppp2r5c Ppp4c Prkaa1 Rpap1 Sae1 Snrpd3 Spcs2 Strn3 Suz12 Tada1 Tpr Ube2s*

The underlined blue tags are hyperlinks to external annotation resources. To follow the link press the **CONTROL** key and simultaneously **CLICK** on the link with the mouse **LEFT** bottom.

Table S4. E3.25 HNC-h-DEGs involved in chromatin-remodeling complexes.

Epigenetic function	Genes	Notes
Polycomb repressive complex 2 (PRC2)	*Suz12*, *Jarid2*	
SWI/SNF	*Actl6a*, *Arid1a*, *Brd8*, *Ino80e*, *Kat5*, *Mbd3*	
NURF	*Smarca1*	Actin dependent regulator of chromatin
DNA demethylation	*Tet1*	Catalyzes the conversion of the modified genomic base 5-methylcytosine (5mC) into 5-hydroxymethylcytosine (5hmC) and plays a key role in active DNA demethylation
	Parp1	Marks genomic sequences that must remain unmethylated, protecting them from methylation. Localizes within the *Dnmt1* promoter protecting its unmethylated state (Zampieri *et al.*, 2009)
Imprinting	*Meg3*	
Histone chaperon	*Ncl*/nucleolin	Mediates nucleosome disruption critical for DNA double-strand break repair (Goldstein *et al.*, 2013)
Chromatin modifier	*Trpm7*	"chanzyme" that codifies an ion channel that modifies histones through cleaving of kinase fragments, that translocate to the nucleus and bind components of chromatin-remodeling complexes, including Polycomb group proteins (Krapivinsky *et al.*, 2014)
	Msh6	Inhibits or removes chromosomal compaction (Edelbrock *et al.*, 2013)
Other chromatin related genes	*Baz2a, Hmgb2, Hmgb3, Mbd3, Myc, Psme4, Satb1, Scmh1,* and *Ssrp1*	

E3.25 HNC-h-DEGs: Highly expressed genes in the E3.25-HNC (E3.25 high-number-of-cell embryos).

Table S5. Description of the intersection members between the E3.25 HNC-h-DEGs and the Oct4-Sox2 interactome.

Gene	Description
Cct3	Chaperonin member of the TCP1 ring complex (TriC) which folds among various proteins actin and tubulin
Hnrnpu	Heterogeneous nuclear ribonucleoprotein U that exerts a global control of alternative splicing by regulating U2 snRNP maturation. Hnrnpu (SAF-A) is involved in the regulation of *Oct4* expression and binds the *Oct4* proximal promoter in ESCs (Vizlin-Hodzic *et al.,* 2011)
Parp1	Safeguards the pluripotent state by occupying key pluripotency genes, notably *Nanog*, *Pou5f1*, *Sox2*, *Dppa3/Stella*, *Tet1* and *Zfp42*, thereby protecting them from progressive epigenetic repression (Roper *et al.*, 2014)
Ssrp1	Encodes a subunit of a the chromatin transcriptional elongation factor FACT, which interacts specifically with histones H2A/H2B to effect nucleosome disassembly and transcription elongation

E3.25 HNC-h-DEGs: Highly expressed genes in the E3.25-HNC (E3.25 high-number-of-cell embryos).

Supplementary references

Edelbrock MA, Kaliyaperumal S, Williams KJ. Structural, molecular and cellular functions of MSH2 and MSH6 during DNA mismatch repair, damage signaling and other noncanonical activities. *Mutat Res.***743-744**, 53-66. Review (2013).

Goldstein M, Derheimer FA, Tait-Mulder J, Kastan MB. Nucleolin mediates nucleosome disruption critical for DNA double-strand break repair. *Proc Natl Acad Sci U S A,* **110**(42), 16874-9 (2013).

Krapivinsky G, Krapivinsky L, Manasian Y, Clapham DE. The TRPM7 chanzyme is cleaved to release a chromatin-modifying kinase. *Cell* **157**(5):1061-72 (2014).

Ohnishi Y, Huber W, Tsumura A, Kang M, Xenopoulos P, Kurimoto K, Oleś AK, Araúzo-Bravo MJ, Saitou M, Hadjantonakis AK, Hiiragi T. Cell-to-cell expression variability followed by signal reinforcement progressively segregates early mouse lineages. *Nat. Cell Biol.* **16**, 27-37 (2014).

Roper SJ, Chrysanthou S, Senner CE, Sienerth A, Gnan S, Murray A, Masutani M, Latos P, Hemberger M. ADP-ribosyltransferases Parp1 and Parp7 safeguard pluripotency of ES cells. *Nucleic Acids Res.* **42**(14):8914-27 (2014).

Vizlin-Hodzic D, Johansson H, Ryme J, Simonsson T, Simonsson S. SAF-A has a role in transcriptional regulation of Oct4 in ES cells through promoter binding. *Cell Reprogram.* **13**(1):13-27 (2011).

Zampieri M, Passananti C, Calabrese R, Perilli M, Corbi N, De Cave F, Guastafierro T, Bacalini MG, Reale A, Amicosante G, Calabrese L, Zlatanova J, Caiafa P. Parp1 localizes within the Dnmt1 promoter and protects its unmethylated state by its enzymatic activity. *PLoS One* **4**(3):e4717 (2009).
